# Supplementary material for: Suppressive Effects of Volatile Compounds from Bacillus spp. on Magnaporthe oryzae Triticum (MoT) Pathotype, Causal Agent of Wheat Blast
Source: Microorganisms. 2023 May 16;11(5):1291. doi: 10.3390/microorganisms11051291 (PMC10221767; doi:10.3390/microorganisms11051291)
Supplement: Supplementary file 1 [file microorganisms-11-01291-s001.zip › microorganisms-2376363-supplementary.pdf]

Supplementary figure S1

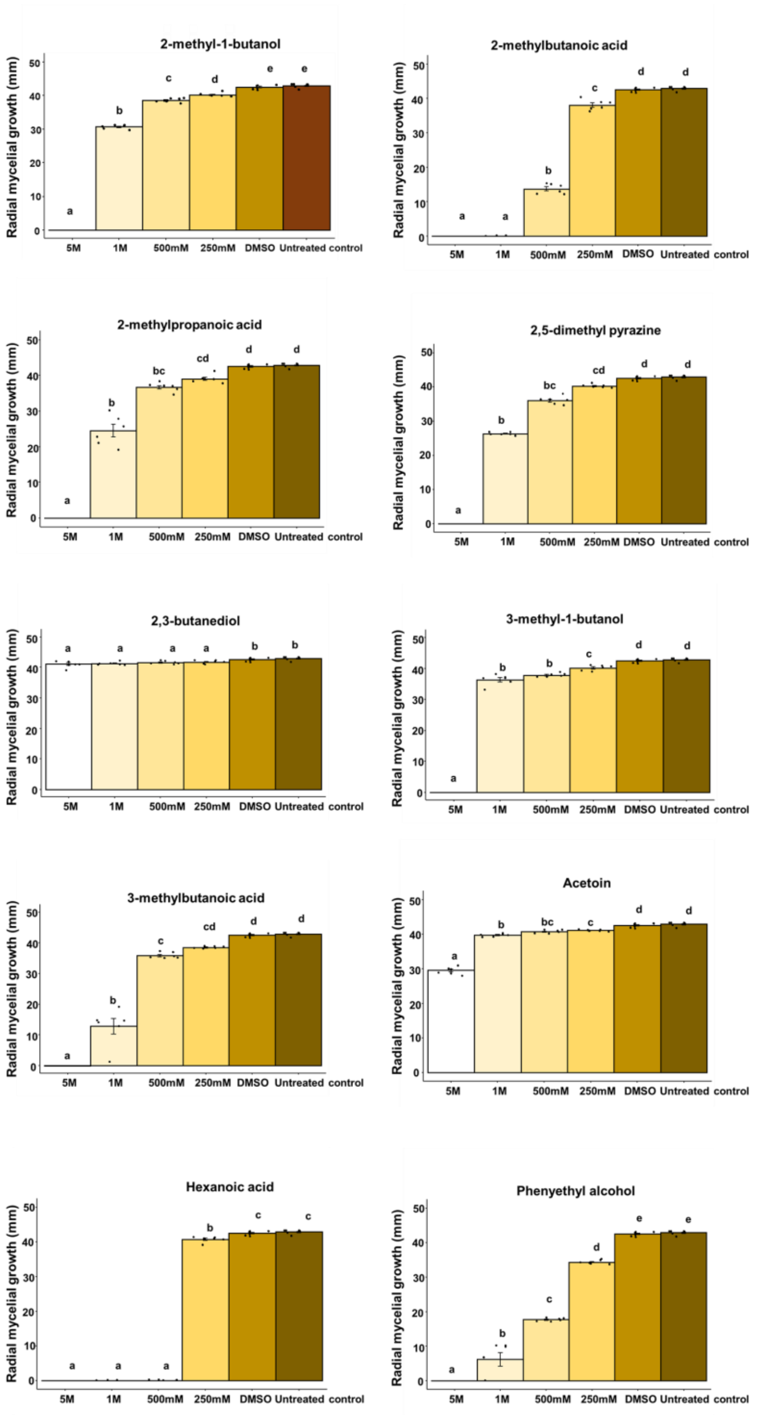

Supplementary figure S1. Assessment of pure VOCs on MoT mycelial growth in upside-down Petri dish assay *in vitro*. Data were recorded 5 d after MoT inoculation at 25˚C (Dunn test; n = 6; $p \leq 0.05$). Each data point represents each replication within the same sampling data set.

Supplementary figure S2

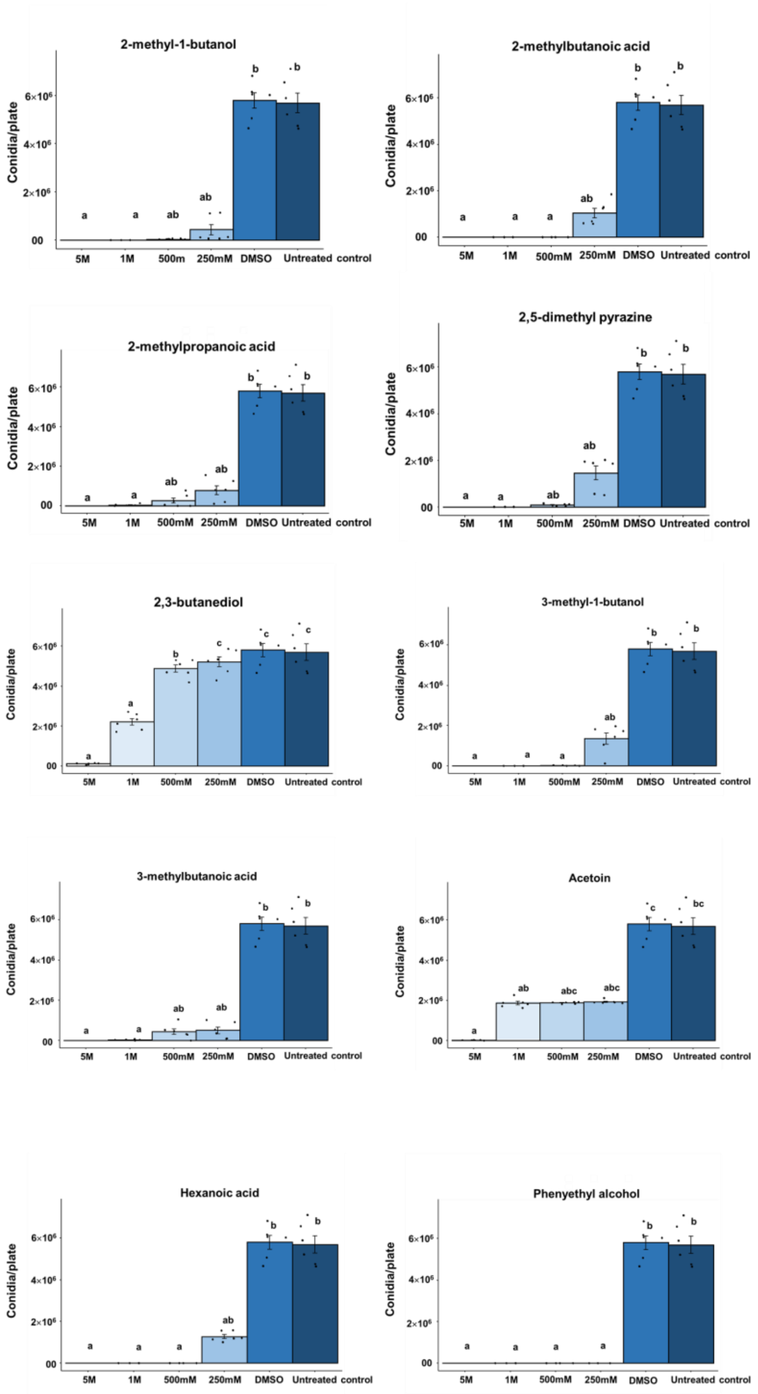

Supplementary figure S2. Assessment of pure VOCs on sporulation of MoT in upside-down Petri dish assay *in vitro*. Data were recorded 5 d after MoT inoculation at 25°C (Dunn test; n = 6; $p \leq 0.05$). Each data point represents each replication within the same sampling data set.

Supplementary figure S3

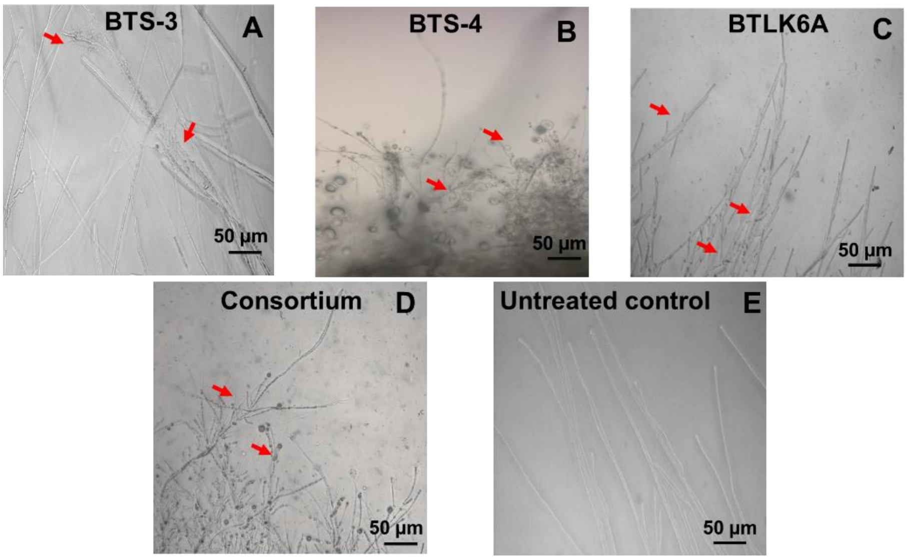

Supplementary figure S3. Alternation of MoT mycelial morphology of representative samples caused by antifungal VOCs from *Bacillus* spp. Pictures were taken at 5 d after MoT inoculation at 25°C (10X magnification). Where, BTS-3: *B. subtilis* BTS-3, BTS-4: *B. amyloliquefaciens* BTS-4, BTLK6A: *B. amyloliquefaciens* BTLK6A, consortium: a mixture of BTS-3, BTS-4 and BTLK6A; and untreated control: water.

Supplementary table S1: Details of purchased pure volatile compounds

Chemical name	CAS number	Molar mass (g/mol)	Company
2-Methylpropionic acid (99%)	79-31-2	88.11	SIGMA, sigma-aldrich Chemie GmbH, Riedstr. 2 D-89555, Steinheim 49 7329 970
3-Methyl-1-butanol (98%)	123-51-3	88.15	SIGMA, sigma-aldrich Chemie GmbH, Riedstr. 2 D-89555, Steinheim 49 7329 970
3-Methylbutanoic acid (99%)	503-74-2	102.13	SIGMA, sigma-aldrich Chemie GmbH, Riedstr. 2 D-89555, Steinheim 49 7329 970
2,3-Butanediol (98%)	513-85-9	90.12	SIGMA, sigma-aldrich Chemie GmbH, Riedstr. 2 D-89555, Steinheim 49 7329 970

2-Methylbutyric acid (98%)	116-53-0	102.132	SIGMA, sigma-aldrich Chemie GmbH, Riedstr. 2 D-89555, Steinheim 49 7329 970
Phenylethyl alcohol (≥99%)	60-12-8	122.167	SIGMA, sigma-aldrich Chemie GmbH, Riedstr. 2 D-89555, Steinheim 49 7329 970
Hexanoic acid (≥98%)	142-62-1	116.16	SIGMA, sigma-aldrich Chemie GmbH, Riedstr. 2 D-89555, Steinheim 49 7329 970
2-Methyl-1-butanol (≥99%)	137-32-6	88.15	SIGMA, sigma-aldrich Chemie GmbH, Riedstr. 2 D-89555, Steinheim 49 7329 970
2,5-Dimethylpyrazine (≥98%)	123-32-0	108.14	SIGMA, sigma-aldrich Chemie GmbH, Riedstr. 2 D-89555, Steinheim 49 7329 970
Acetoin (95%)	513-86-0	88.105	SIGMA, sigma-aldrich Chemie GmbH, Riedstr. 2 D-89555, Steinheim 49 7329 970

Supplementary table S2: Identification and quantification of bacterial VOCs

**Compound**	**RI (L)**	**RI (E)**	**BTS-3**	**BTS-4**	**BTLK6A**	**Consortium**
			ng/h/ml	ng/h/ml	ng/h/ml	ng/h/ml
*Acetoin	706.0	706.5	-	0.911	-	-
*1-Butanol, 3-methyl-	734.0	731.8	-	0.181	-	0.015
*Pyrazine, 2,5-dimethyl-	911.0	912.4	1.081	0.026	0.600	0.825
*1-Butanol, 2-methyl-	736.0	736.8	-	0.023	-	-
*Propanoic acid, 2-methyl-	793.0	797.4	0.152	0.047	0.038	0.027
*2,3-Butanediol	782.0	783.3	-	0.429	-	0.004
*Butanoic acid, 3-methyl-	866.0	868.5	0.324	0.303	0.465	0.058
*Butanoic acid, 2-methyl-	886.2	883.3	0.580	0.667	0.537	0.081
*Hexanoic acid	993.0	992.3	0.009	0.009	-	-

*Phenyl ethyl alcohol	1120.0	1123.1	-	0.023	-	0.004
2-Heptanone	895.0	893.0	0.011	0.020	-	0.011
Heptanal	904.0	903.8	-	0.009	-	-
1-Hexene, 4,5-dimethyl-	970.0	900.0	-	-	-	0.049
2-Heptanone, 6-methyl-	932.0	900.5	0.023	-	0.033	0.023
Benzaldehyde	921.0	900.6	0.005	-	-	0.003
2-Heptanone, 5-methyl-	974.0	999.0	0.025	0.009	0.018	0.005
2 Methyl pyrazine	826.0	820.0	-	0.062	-	-
Pyrazine, 2-ethyl-3-methyl-	999.0	991.1	0.003	-	-	-
Octanal	1003.0	1004.6	-	0.013	-	-
Pyrazine, trimethyl-	989.0	1000.0	0.092	0.014	0.040	0.044
2-Acetylthiazole	1014.0	1022.0	0.010	0.005	0.009	0.007
1-Hexanol, 2-ethyl-	1029.0	1030.3	0.042	0.020	0.015	0.004
Benzyl alcohol	1035.7	1037.2	-	0.010	-	0.011
Undecane	1000.0	1000.0	0.007	0.005	-	-
Acetophenone	1065.0	1071.5	0.012	0.011		0.006
Phenol, 2-methoxy-	1096.0	1093.0	-	0.125	-	-
2-Nonanol	1102.0	1100.0	-	0.017	-	-
Nonanal	1104.0	1105.0	0.015	0.025	-	0.006
2-Decanone	1191.0	1100.0	-	0.004	-	-
1-Decene	991.0	993.0	-	0.005	-	-
Dodecane	1200.0	1200.0	-	0.003	0.003	-
Decanal	1205.0	1206.9	-	0.003	-	-
Benzothiazole	1221.0	1201.0	0.008	0.004	0.007	0.004
Nonanoic acid	1272.0	1263.4	-	0.018	-	-

2-Undecanone	1294.0	1295.3	-	0.014	-	-
1-Pentanol	744.1	741.9	-	-	-	0.008
Hexanal	811.0	810.7	0.012	0.091	-	-
3-Pentanol	814.0	800.0	-	0.055	-	-
2-Hydroxy-3-pentanone	821.0	800.2	-	0.049	-	-
Pyrazine, 2-methyl-	853	859.3	-	0.014	-	0.011
Diethyl acetic acid	885	846.7	-	1.083	-	-

Where, BTS-3= *B. subtilis* BTS-3; BTS-4= *B. amyloliquefaciens* BTS-4; BTLK6A= *B. amyloliquefaciens* BTLK6A; consortium=a mixture of BTS-3, BTS-4 and BTLK6A; *= Gold identified by using pure compound; RT = Retention time; RI (L) = Retention Index (Literature); RI (E) = Retention Index (Experimental)

Supplementary table S3: Effect of pure VOCs to suppress MoT *in vitro*

Compound	Concentration	Mycelial radial growth (mm)	Total conidia/plate
2-Methyl-1-butanol	5M	0.0 ± 0.0	0.0
	1M	30.7 ± 0.2	0.0
	500mM	38.5 ± 0.2	3.2×10^{4}
	250mM	40. 1 ± 0.1	4.2×10^{5}
2-Methylbutanoic acid	5M	0.0 ± 0.0	0.0
	1M	0.0 ± 0.0	0.0
	500mM	13.7 ± 1.5	0.0
	250mM	38.0 ± 0.6	1.1×10^{6}
2-Methylpropanoic acid	5M	0.0 ± 0.0	0.0
	1M	24.0 ± 1.7	4.2×10^{4}
	500mM	36.7 ± 0.4	2.6×10^{5}

	250mM	39.0 ± 0.4	7.8×10^{5}
2,5-Dimethyl pyrazine	5M	0.0 ± 0.0	0.0
	1M	26.33 ± 0.2	0.0
	500mM	36.0 ± 0.4	8.6×10^{4}
	250mM	40.2 ± 0.1	1.5×10^{6}
2,3-Butanediol	5M	41.0 ± 0.4	1.1×10^{5}
	1M	41.2 ± 0.2	2.2×10^{6}
	500mM	41.5 ± 0.2	4.8×10^{6}
	250mM	41.7 ± 0.2	5.2×10^{6}
3-Methyl-1-butanol	5M	0.0 ± 0.0	0.0
	1M	36.33 ± 0.7	0.0
	500mM	37.8 ± 0.3	1.5×10^{4}
	250mM	40.2 ± 0.4	1.4×10^{6}
3-Methylbutanoic acid	5M	0.0 ± 0.0	0.0
	1M	12.8 ± 2.5	3.3×10^{4}
	500mM	35. 5 ± 0.4	4.4×10^{5}
	250mM	38.5 ± 0.2	5.0×10^{5}
Acetoin	5M	29.5 ± 0.4	2.7×10^{4}
	1M	39.7 ± 0.2	1.7×10^{6}
	500mM	40.7 ± 0.2	1.8×10^{6}
	250mM	41.0 ± 0.0	1.9×10^{6}
Hexanoic acid	5M	0.0 ± 0.0	0.0
	1M	0.0 ± 0.0	0.0
	500mM	0.0 ± 0.0	0.0
	250mM	40.7 ± 0.3	1.3×10^{6}
Phenylethyl alcohol	5M	0.0 ± 0.0	0.0
	1M	6.2 ± 2.0	0.0
	500mM	17. 6 ± 0.2	0.0
	250mM	34.3 ± 0.2	0.0
DMSO	-	42.5 ± 0.2	5.8×10^{6}
Untreated control	-	42.8 ± 0.1	5.7×10^{6}
